# Decomposition of economic inequalities in dental caries among Iranian schoolchildren

**DOI:** 10.1371/journal.pone.0306778

**Published:** 2024-08-22

**Authors:** Maryam Khoramrooz, Seyed Mohammad Mirrezaie, Mohammad Hassan Emamian, Ali Dadgari, Hassan Hashemi, Akbar Fotouhi

**Affiliations:** 1 Department of Health Management and Economics, School of Public Health, Hamadan University of Medical Sciences, Hamadan, Iran; 2 Modeling of Noncommunicable Diseases Research Center, Hamadan University of Medical Sciences, Hamadan, Iran; 3 Center for Health Related Social and Behavioral Sciences Research, Shahroud University of Medical Sciences, Shahroud, Iran; 4 Ophthalmic Epidemiology Research Center, Shahroud University of Medical Sciences, Shahroud, Iran; 5 School of Nursing and Midwifery, Shahroud University of Medical Sciences, Shahroud, Iran; 6 Noor Ophthalmology Research Center, Noor Eye Hospital, Tehran, Iran; 7 Department of Epidemiology and Biostatistics, School of Public Health, Tehran University of Medical Sciences, Tehran, Iran; University of Minnesota School of Dentistry, UNITED STATES OF AMERICA

## Abstract

**Introduction:**

Monitoring social inequalities in dental caries is crucial for establishing priorities in oral health systems. This study aimed to assess economic inequalities in dental caries and its contributing factors among Iranian schoolchildren.

**Methods:**

Data were obtained from the first phase of the Shahroud Schoolchildren Eye Cohort Study in 2015. A total of 4992 children aged 6–12 years old were included in the analysis. Dental examinations were conducted following the diagnostic methods and standards of the Oral Health Examination Survey, as recommended by the World Health Organization. The concentration index (C) was utilized to assess economic inequalities in dental caries. Additionally, the decomposition of C was employed to explain the determinants of the measured inequalities.

**Results:**

In total, 71.4% of the schoolchildren had dental caries in primary dentition (dft≥1), and 41.6% of the schoolchildren had dental caries in permanent dentition (DMFT≥1). The Cs of dft≥1, primary decayed teeth (pdt≥1), and permanent missing teeth (PMT≥1) were -0.136 (95% CI: -0.167, -0.104), -0.164 (95% CI: -0.194, -0.134), and -0.208 (95% CI: -0.262, -0.153), respectively, which indicates their more concentration among low-economic children. Conversely, pft≥1 and PFT≥1 had Cs of 0.327 (95% CI: 0.292, 0.361) and 0.218 (95% CI: 0.179, 0.256), showing more concentration among high-economic children. Basic health insurance coverage and age were the main contributors that explained 28.6% and 19.2% of the economic inequality in dft≥1, and 25.7% and 16.6% of the pdt≥1 inequality, respectively. Economic status, residence in rural areas, mother education, father education, and age were the main contributors to the measured inequality in pft≥1 by 80.5% and 26.5%, 21.9%, 22%, and -18.3%, respectively. Economic status, having a housekeeper mother, residence in rural areas, having basic health insurance coverage, mother education, and father education positively contributed to the measured inequality in PMT≥1 by 45.4%, 42.4%, 37.8%, 35.1%, 21.3%, and 15.2%, respectively, while age had a negative contribution of -19.3%. For PFT≥1, economic status, age, and father education accounted for 76%, 25.4%, and 20.3% of the measured inequality, respectively.

**Conclusion:**

Pro-rich economic inequalities were observed in children’s primary and permanent teeth caries. Thus, government interventions to reduce these inequalities should aim to expand the coverage of basic and supplementary health insurance in line with increasing the coverage of dental health costs in these plans, training and providing access to required dental health services for low-socioeconomic children, including the poor, rural, and those who have low-educated parents and a housekeeper mother, especially at younger ages.

## 1. Introduction

Dental caries is a global public health concern and the most common non-communicable disease worldwide [1]. The 2022 World Health Organization (WHO) global report on oral health indicates that globally more than 2 billion people are affected by dental caries in permanent teeth, with 514 million children experiencing caries in their primary teeth [2]. In 2019, sociodemographic inequality was responsible for 64.6 million (3.2%) cases of prevalent permanent teeth caries and 62.9 million (12.1%) cases of primary teeth caries globally [3]. Dental caries has been associated with a decreased quality of life for children and their families [4].

A multinational study revealed that socioeconomic inequality can have a negative impact on self-rated oral health and is closely linked to untreated dental caries among adult participants [5]. Research conducted in the north of England confirmed a strong association between tooth decay and social deprivation. Furthermore, it was determined that the introduction of water fluoridation has significantly decreased tooth decay in 12-year-old children and reduced socioeconomic inequalities in dental health [6]. However, another study indicated that the interactions among stores, diet, and sugar consumption may be the most crucial neighborhood-based factors contributing to tooth decay, regardless of socioeconomic status [7].

The significant impact of social, environmental, behavioral, and biological factors on early childhood caries is well-documented [8]. Socioeconomic and environmental factors, along with family size and parental employment and education, have an impact on the incidence of caries [9, 10]. Moreover, socioeconomic factors play a vital role in determining the range of services included in primary oral health care. Having access to public dental care services can help decrease the prevalence of dental caries in marginalized populations [11]. Another known risk factor for caries is the frequency of sweet intake. Consuming an unhealthy diet, including high-calorie foods, has been identified as a key factor contributing to the rising prevalence of dental caries [12].

Studies in Iran indicate a link between socioeconomic factors and children’s dental caries. Kazemi Karyani et al. demonstrated in their research that mothers’ education, female gender, and dental visits were factors influencing permanent tooth decay in children aged 12 to 15 years [13]. Ghasemianpour et al. also found a relationship between social and economic factors and tooth decay in children. Their study revealed a positive association between children’s tooth decay and residing in rural areas, the consumer price index, and the Gini coefficient, as well as a negative association with parents’ education and GDP [14]. Amanlou et al. observed a positive link between tooth decay in children aged 3 to 6 years and parents’ education [15]. Yousefi et al. identified a positive relationship between children’s tooth decay and living in rural areas [16].

Monitoring social inequalities in dental caries is essential for understanding the need for preventive measures and oral health care priorities. Several studies in Iran have explored the socioeconomic factors related to children’s dental caries [13–17]. However, there has been limited focus on quantifying socioeconomic inequalities in children’s tooth decay and the utilization of dental treatment services. The present study aimed to investigate and decompose the economic inequalities associated with dental caries in children’s primary and permanent dentition, focusing on a large representative sample from Shahroud, northeastern Iran. By determining the contribution of demographic and socioeconomic factors to the measured economic inequalities in children’s dental caries, we aim to provide new insights and recommendations for addressing these inequalities through targeted government interventions.

## 2. Methods

### 2.1. Source of data and study variables

A cross-sectional analysis of data from the first phase of the Shahroud Schoolchildren Eye Cohort Study (SSCECS) was performed. The cohort study was launched in 2015, originally intended to be conducted in urban and rural areas of Shahroud County, northeast Iran. In rural areas, a census method was utilized for student recruitment due to the small population. Therefore, the probability of selecting each student in rural areas and the sampling weight was equal to one. Sampling in urban areas followed a cluster random sampling design, where each classroom served as a cluster, and 200 out of 473 clusters were systematically chosen. Consequently, the probability of selecting each student in urban areas stood at 0.4228 (200/473), with a sampling weight of 2.365 (1/0.4228). Further details regarding the sampling procedure can be found elsewhere [18, 19].

Out of 6624 children selected, 5620 (84.8%) participated in the SSCECS. The participants were 1154 students (95.1%) of the 1214 rural students and 4466 students (82.6%) of the 5410 urban students. Following parental consent, dental examinations were conducted on a specified date at the examination site. These examinations involved evaluating tooth surfaces to identify tooth decay using diagnostic methods and standards from the Oral Health Examination Survey, as suggested by the WHO [20]. More details on the examination methods and calculation of dental caries indicators have been previously outlined [21]. The guideline of the WHO [20] was used to train dentists. All photos in this guideline depict the outcomes that are related to oral and dental health. A joint review was conducted to establish a consistent approach in line with these guidelines. Additionally, parents of the participating children completed a questionnaire on demographic characteristics and household assets. Following the removal of any missing data, the study analysis was conducted on data from 4992 children.

The DMFT was used as the outcome variable for permanent teeth caries, calculated as the sum of untreated or recurrent cavities named decayed teeth (D-component), missing teeth due to extraction (M-component), and filled teeth with no caries (F-component). Similarly, dft was used as the outcome variable for children’s primary teeth caries, defined as the sum of primary decayed teeth (d-component) and filled teeth with no caries (f-component). The outcome variables of DMFT and dft were dichotomized as having decayed (D/d), missing (M), or filled (F/f) permanent/primary teeth into “yes” (DMFT/dft≥1) or “no” (DMFT/dft = 0). Similarly, we calculated the PDT≥1/pdt≥1 as having decayed permanent/primary teeth, PMT≥1 as having missing permanent teeth (M), and PFT≥1/pft≥1 as having filled permanent/primary teeth.

The DMFT index only represents the mean of permanent tooth decay in children, including those who are caries free and those with a high level of tooth decay. In an effort to identify children with the highest number of dental caries [22], we followed the methodology outlined by the WHO Collaborating Centre in Sweden, in calculating the significant caries index (SiC). The SiC was computed as the average DMFT of one-third of the children with the most severe caries scores [23]. In this study, to highlight the prevalence of the highest caries burden, we introduced a variable called significant caries (SC≥1). Children who fell within the upper third of the DMFT index were assigned a code of 1, while the remaining children were assigned a code of 0.

Households’ economic status was calculated by performing a principal component analysis (PCA) [24] on home assets, including car, motorcycle, TV, LCD, bathroom, vacuum cleaner, washing machine, refrigerator, freezer, computer, telephone, internet, microwave, and dishwasher. The households were then categorized into five economic quintiles, according to their asset index score. Other variables considered in the study included children’s gender (boy, girl), age (6, 7, 8, 9, 10, 11, and 12 years old), rural residency (yes, no), father education (<5, 5–12, and >12 years), mother education (<5, 5-12T and >12 years), father employment status (employed, unemployed or retired, other), mother employment status (housekeeper, other), type of school (public, private), overweight/obesity (yes, no), basic health insurance coverage (yes, no), and having supplementary health insurance (yes, no). The BMI for age Z-score was determined following the World Health Organization growth reference. Students with a Z-score >1 were classified as overweight/obese [25, 26].

### 2.2. Inequality measurement

We utilized the concentration index (C) to assess the economic inequality of dental caries. The index was computed based on the concentration curve, showing the cumulative share of dental caries indices compared to the cumulative share of children’s economic status.

The C value was determined as twice the area between the concentration curve and the line of equality, with the index ranging from -1 to +1. A negative (positive) C value indicates that the dental caries index is more concentrated among low- (high-) economic children when the concentration curve is below (above) the line of equality. A C of zero is observed when the concentration curve aligns with the line of equality, indicating no economic inequality in the distribution of the dental caries index. The concentration index was calculated using the formula below:

$$\text{c}=\;2\text{Cov}\left({\text{y}}_{\text{i}},{\text{r}}_{\text{i}}\right)/\mu$$ (1)

Cov is the covariance, *y*_*i*_ is the index of dental caries for *i*-th children, *r*_*i*_ is the fractional rank of *i*-th children in the distribution of households’ economic status, and *μ* is the mean of dental health index. As the outcome variables were binary, we normalized the C using the Wagstaff approach, [27] as follows:

$$C=\frac{c}{\left(1-\mu \right)}$$ (2)

In the above equation, C is the standardized concentration index. If there is no inequality in the dental health index, the value is zero, and if there is more concentration of index among poor (rich) children, the value is negative (positive).

### 2.3. Decomposition of inequality

After measuring the inequality in children’s dental caries indices, our next step was to determine the factors that had main contributions to the measured inequalities. To do this, we used the decomposition method that was first proposed by Wagstaff et al [28]. This approach entailed decomposing the inequalities observed in children’s dental health indices into two key components using the specified formula:

$$C=\sum_{k}\left(\frac{\beta_{k}{\bar{X}}_{k}}{\mu }\right)C_{k}+\frac{C_{e}}{\mu }$$ (3)

The first component ($\sum_{k}\left(\frac{\beta_{k}{\bar{X}}_{k}}{\mu }\right)C_{k}$) is the part of the inequality that is attributable to the explanatory variables and includes the summed contribution of the study covariates to the measured inequality. The second part ($\frac{C_{e}}{\mu }$) includes the residual component of inequality that cannot be accounted for by the study covariates.

To conduct the decomposition analysis, we first estimated the marginal effects of the study covariates (*β*_*k*_) through logistic regression. Next, we calculated the elasticity of the outcome variable concerning changes in the study covariates ($\frac{\beta_{k}{\bar{X}}_{k}}{\mu }$) by multiplying the marginal effects by the mean of the covariates (${\bar{X}}_{k}$) and dividing the result by the mean of the outcome variable (*μ*). Subsequently, we determined the contribution of each covariate to the observed inequality in dental health indices as the product of the elasticity in the concentration index of that variable (*C*_*k*_). Additionally, we computed the residual component ($\frac{C_{e}}{\mu }$) by subtracting $\sum_{k}\left(\frac{\beta_{k}{\bar{X}}_{k}}{\mu }\right)C_{k}$ from C.

In estimating the marginal effects, we employed the Variance Inflation Factor (VIF) to assess potential multicollinearity among the covariates. The average VIF was 1.22, the highest VIF was 1.69, and all individual tolerance statistics were >0.59, indicating no notable multicollinearity among the explanatory variables. According to established guidelines, VIF values below a specific threshold are deemed acceptable to ensure dependable regression coefficient estimates. VIF values close to or exceeding 5 may indicate significant correlation among predictors, prompting concerns about multicollinearity [29].

All estimates of the prevalence, concentration indices, and confidence intervals were adjusted for the effects of the sampling frame (strata, clusters, and sampling weights). Statistical analyses were conducted using Stata software (package V.14.0). P<0.05 was considered to be statistically significant.

### 2.4. Ethics approval

All methods of the study were carried out by relevant guidelines and regulations. The protocol of the study was approved by the Ethics Committee of Shahroud University of Medical Sciences (Ethics code: 100/108054). Students participate in the study willingly. Written informed consent was signed by students’ parents.

## 3. Results

Among the 5620 participants in the first phase of the SSCECS, dental examination data were available for 5577 students. However, 585 participants were excluded from the final analysis because of missing data in insurance status or parents’ education variables, resulting in a final analysis of data from 4992 children. Of the study subjects, 2334 (46.6%) were girls, 3965 (90.1%) resided in urban areas, 2776 (55.9%) had a father with 5–12 years of education, 2836 (58.1%) had a mother with 5–12 years of education, 4320 (87.2%) had an employed father, 4190 (83.2%) had a housekeeper mother, 4585 (90.8%) attended public schools, 3833 (75.7%) were overweight/obese, 4918 (92.2%) were covered by a basic health insurance scheme, and 937 (21.1%) had supplementary health insurance coverage. The largest proportion of children, 959 (19.2%), belonged to the 9-year-old age group, while the 6-year-old age group had the smallest proportion of 202 (3.6%) (Table 1).

**Table 1:** Summary statistics for schoolchildren by dental caries indices in Shahroud, Iran, 2015.

Characteristics		Total sample n (%)	dft≥1 n (%)	pdt≥1 n (%)	pft≥1 n (%)	DMFT≥1 n (%)	PDT≥1 n (%)	PMT≥1 n (%)	PFT≥1 n (%)	SC≥1 n (%)
Gender	Boy	2658 (53.44)	1933 (72.43)	1891 (70.64)	317 (13.41)	1055 (39.45)	950 (35.79)	87 (2.42)	147 (5.69)	833 (31.14)
	Girl	2334 (46.56)	1641 (70.14)	1590 (67.68)	291 (14.04)	1033 (44.04)	913 (38.77)	67 (2.34)	187 (8.59)	831 (35.58)
***P-value***		***-***	***0*.*548***	***0*.*432***	***0*.*739***	***0*.*070***	***0*.*213***	***0*.*878***	***0*.*003***	***0*.*060***
Age (Years)	6	202 (3.62)	193 (95.80)	193 (95.80)	20 (12.19)	24 (11.44)	17 (9.22)	8 (2.12)	3 (1.16)	16 (7.15)
	7	739 (14.78)	706 (95.55)	693 (93.55)	143 (21.82)	125 (16.21)	104 (13.96)	12 (0.96)	16 (2.02)	85 (10.94)
	8	909 (18.18)	856 (94.24)	834 (91.49)	191 (23.73)	309 (33.15)	265 (28.73)	23 (1.72)	47 (4.88)	234 (25.21)
	9	959 (19.21)	838 (87.73)	817 (85.32)	142 (16.59)	428 (44.15)	387 (39.98)	27 (1.90)	56 (6.15)	334 (34.39)
	10	805 (16.11)	543 (67.59)	527 (65.33)	62 (8.67)	389 (47.81)	345 (42.17)	32 (3.46)	67 (8.48)	312 (38.25)
	11	825 (16.93)	339 (40.94)	323 (38.80)	40 (5.37)	472 (57.56)	429 (52.18)	32 (3.13)	83 (10.91)	387 (47.47)
	12	553 (11.17)	99 (17.57)	94 (16.55)	10 (2.04)	341 (61.07)	316 (55.98)	20 (3.60)	67 (13.17)	296 (53.21)
***P-value***		***-***	***<0*.*001***	***<0*.*001***	***<0*.*001***	***<0*.*001***	***<0*.*001***	***0*.*018***	***<0*.*001***	***<0*.*001***
Place of residence	Urban	3965 (90.13)	2821 (71.15)	2729 (68.83)	599 (15.11)	1640 (41.36)	1469 (37.18)	85 (8.28)	292 (7.36)	1312 (33.09)
	Rural	1027 (9.87)	753 (73.32)	752 (73.22)	9 (0.88)	448 (43.62)	394 (38.36)	69 (1.74)	42 (4.09)	352 (34.27)
***P-value***		***-***	***0*.*603***	***0*.*297***	***<0*.*001***	***0*.*503***	***0*.*687***	***<0*.*001***	***0*.*007***	***0*.*702***
Father education (Years)	<5	1009 (17.39)	707 (69.12)	704 (68.73)	28 (3.59)	471 (47.24)	430 (43.24)	53 (4.36)	40 (4.55)	392 (39.40)
	5–12	2776 (55.85)	2004 (72.01)	1970 (70.65)	276 (11.07)	1173 (42.22)	1062 (38.41)	85 (2.30)	175 (6.51)	917 (33.05)
	>12	1207 (26.77)	863 (71.47)	807 (66.72)	304 (25.77)	444 (36.58)	371 (30.67)	16 (1.26)	119 (9.76)	355 (29.51)
***P-value***		-	0.481	***0*.*179***	***<0*.*001***	***<0*.*001***	***<0*.*001***	***<0*.*001***	***<0*.*001***	***<0*.*001***
Mother education (Years)	<5	1218 (20.98)	864 (70.34)	859 (69.80)	42 (4.36)	538 (45.15)	498 (41.51)	50 (3.60)	55 (5.33)	431 (36.19)
	5–12	2836 (58.10)	2051 (72.13)	2007 (70.43)	311 (12.08)	1209 (42.29)	1072 (37.81)	95 (2.50)	186 (6.69)	965 (33.85)
	>12	938 (20.92)	659 (70.24)	615 (65.46)	255 (27.59)	341 (36.06)	293 (31.09)	9 (8.53)	93 (9.73)	268 (28.44)
***P-value***		***-***	***0*.*607***	***0*.*116***	***<0*.*001***	***0*.*003***	***<0*.*001***	***0*.*001***	***<0*.*001***	***0*.*005***
Father employment status	Employed	4320 (87.18)	3128 (72.32)	3045 (70.17)	556 (14.38)	1795 (41.28)	1596 (36.78)	130 (2.35)	291 (7.06)	1415 (32.61)
	Unemployed/retired	475 (9.09)	312 (64.01)	304 (62.01)	41 (10.11)	205 (43.32)	187 (39.54)	15 (2.31)	33 (7.82)	171 (36.40)
	Other	197 (3.73)	134 (66.88)	132 (65.67)	11 (6.70)	88 (44.48)	80 (40.66)	9 (3.37)	10 (4.69)	78 (39.44)
***P-value***		***-***	***<0*.*001***	***0*.*001***	***<0*.*003***	***0*.*572***	***0*.*395***	***0*.*627***	***0*.*477***	***0*.*086***
Mother employment status	Housekeeper	4190 (83.17)	3018 (71.80)	2946 (69.85)	471 (12.73)	1760 (41.74)	1570 (37.39)	142 (2.62)	266 (6.69)	1412 (33.57)
	Other	802 (16.83)	556 (69.18)	535 (66.35)	137 (18.51)	328 (40.80)	293 (36.15)	12 (1.23)	68 (8.80)	252 (31.39)
		***-***	***0*.*268***	***0*.*134***	***0*.*001***	***0*.*657***	***0*.*548***	***0*.*013***	***0*.*035***	***0*.*256***
Economic quintiles	1^st^	971 (15.54)	750 (76.45)	748 (76.16)	26 (3.63)	376 (39.47)	342 (36.27)	43 (4.01)	27 (2.85)	296 (31.73)
	2^nd^	996 (19.08)	756 (76.50)	752 (76.09)	60 (7.01)	422 (41.05)	384 (37.97)	40 (2.56)	48 (5.10)	332 (32.39)
	3^rd^	1074 (22.39)	771 (72.35)	747 (69.91)	140 (13.98)	473 (43.44)	418 (38.45)	36 (2.66)	77 (7.17)	372 (33.90)
	4^th^	973 (21.13)	678 (70.19)	651 (67.29)	167 (17.90)	436 (44.41)	386 (39.16)	22 (1.93)	86 (8.88)	355 (36.07)
	5^th^	978 (21.85)	619 (63.37)	583 (59.63)	215 (22.37)	381 (38.91)	333 (33.92)	13 (1.23)	96 (9.81)	309 (31.48)
***P-value***		***-***	***<0*.*001***	***<0*.*001***	***<0*.*001***	***0*.*089***	***0*.*135***	***0*.*001***	***<0*.*001***	***0*.*201***
Type of school	Public	4585 (90.75)	3301 (71.80)	3225 (69.91)	531 (13.17)	1944 (42.22)	1737 (37.81)	148 (2.48)	300 (6.91)	1545 (33.61)
	Private	407 (9.25)	273 (67.08)	256 (62.90)	77 (18.92)	144 (35.38)	126 (30.96)	6 (1.47)	34 (8.35)	119 (29.24)
***P-value***		***-***	***0*.*409***	***0*.*230***	***0*.*034***	***0*.*104***	***0*.*101***	***0*.*171***	***0*.*403***	***0*.*270***
Overweight/obesity	No	3833 (75.70)	2880 (75.11)	2804 (72.85)	480 (14.31)	1625 (42.24)	1450 (37.75)	138 (2.60)	251 (6.88)	1298 (33.78)
	Yes	1159 (24.30)	694 (59.69)	677 (58.10)	128 (11.81)	463 (39.53)	413 (35.40)	26 (1.73)	83 (7.55)	366 (31.43)
***P-value***		***-***	***<0*.*001***	***<0*.*001***	***0*.*053***	***0*.*178***	***0*.*185***	***0*.*063***	***0*.*437***	***0*.*165***
Basic health insurance	No	74 (0.84)	59 (79.77)	59 (79.77)	0	27 (33.92)	23 (29.36)	7 (7.99)	0	23 (27.80)
	Yes	4918 (99.16)	3515 (71.29)	3422 (69.17)	608 (13.82)	2061 (41.65)	1840 (37.25)	147 (2.34)	334 (7.10)	1641 (33.25)
***P-value***		***-***	***0*.*160***	***0*.*085***	***-***	***0*.*173***	***0*.*142***	***0*.*001***	***-***	***0*.*372***
Supplementary health insurance	No	4055 (78.92)	2913 (71.53)	2857 (69.93)	422 (12.02)	1695 (41.49)	1513 (37.13)	137 (2.57)	250 (6.52)	1343 (32.91)
	Yes	937 (21.08)	661 (70.73)	624 (66.74)	186 (20.00)	393 (41.95)	350 (37.3)	17 (1.71)	84 (9.00)	321 (34.31)
***P-value***		***-***	***0*.*741***	***0*.*181***	***<0*.*001***	***0*.*836***	***0*.*905***	***0*.*130***	***0*.*017***	***0*.*505***

dft: Primary decayed and filled teeth, pdt: Primary decayed teeth, pft: Primary filled teeth, DMFT: Permanent decayed, missing, and filled teeth, PDT: Permanent decayed teeth, PMT: Permanent missing teeth, PFT: Permanent filled teeth, SC: Significant caries

The prevalence of dft≥1 decreased with age from 95.8% in 6-year-old children to 17.6% in their 12-year-old counterparts. dft≥1 was more prevalent among children whose fathers were employed than the children whose fathers were unemployed/retired or have the other employment status. The prevalence of dft≥1 decreased with an improvement of the household’s economic status from 76.5% in the 1st quintile to 63.4% in the 5th quintile. Also, dft≥1 was more prevalent among non-obese/overweight children than their obese/overweight counterparts (75.1% vs. 59.7%). Similar patterns were observed for pdt≥1. The distribution of other dental caries indices was provided in Table 1.

**Fig 1:**
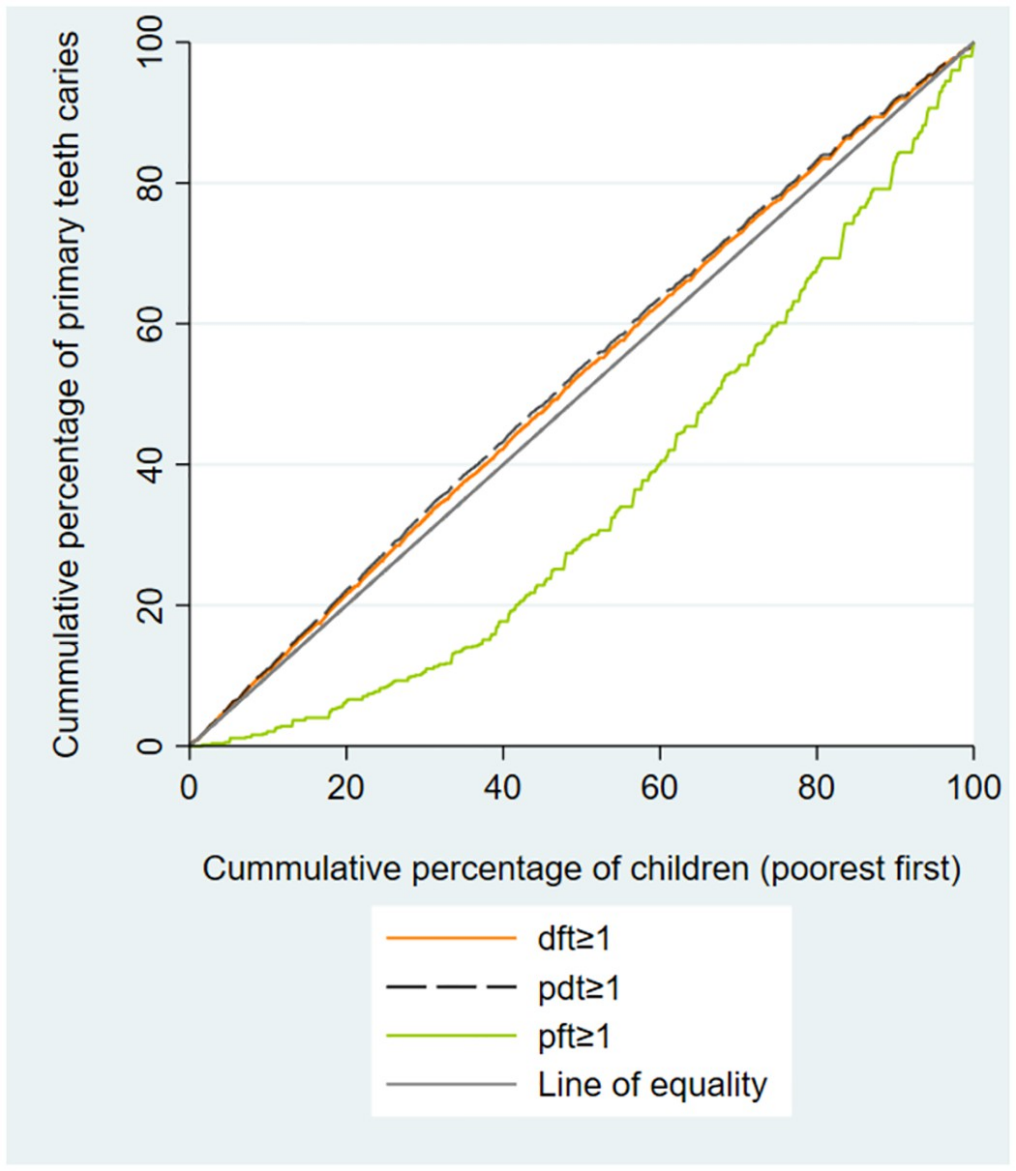
Concentration curves for primary decayed and filled teeth (dft), primary decayed teeth (pdt), and primary filled teeth (pft) indices, among schoolchildren in Shahroud, Iran, 2015.

**Fig 2:**
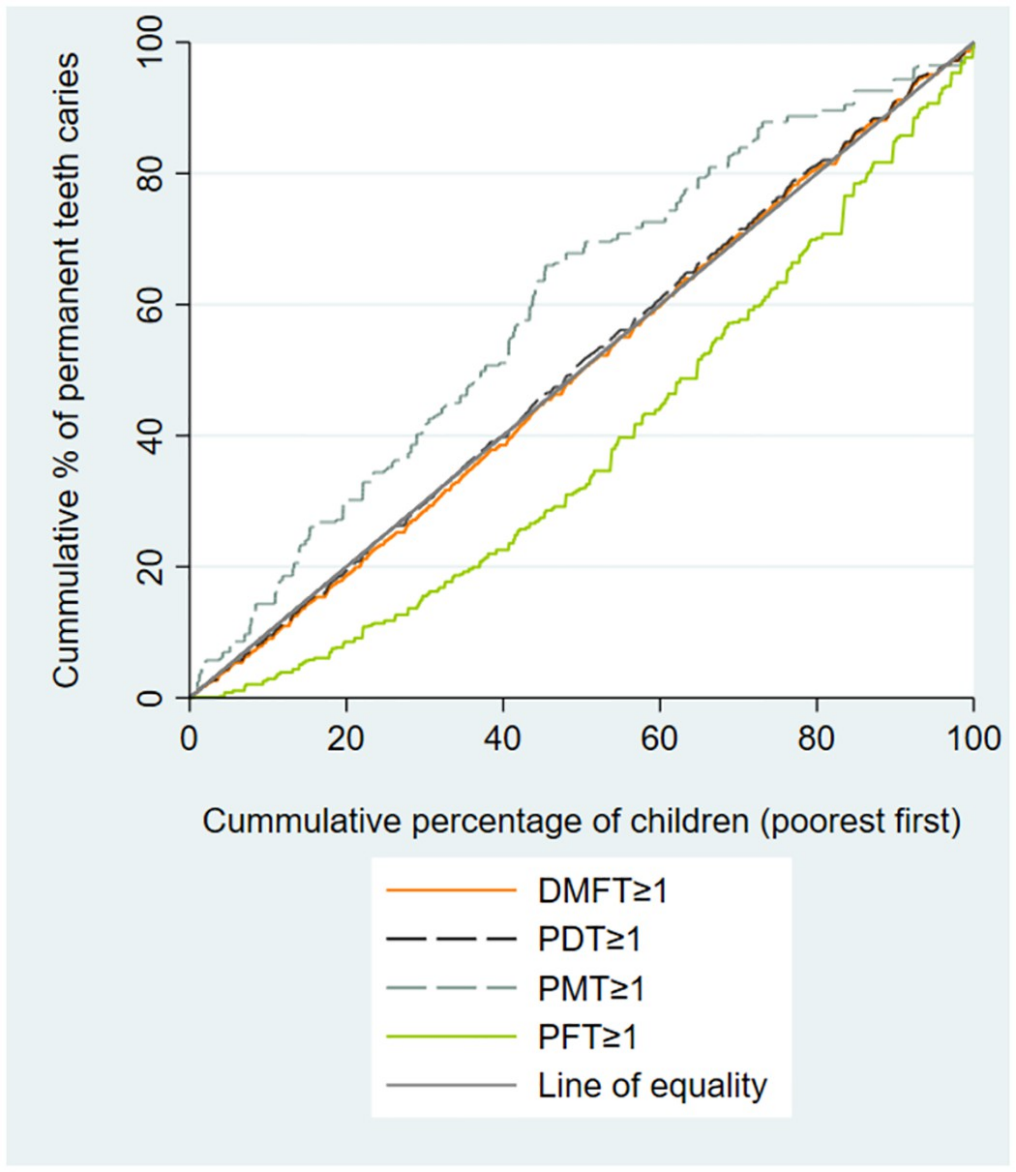
Concentration curves for permanent decayed, missed, and filled teeth (DMFT), permanent decayed teeth (PDT), permanent missing teeth (PMT), and permanent filled teeth (PFT) indices, among schoolchildren in Shahroud, Iran, 2015.

**Fig 3:**
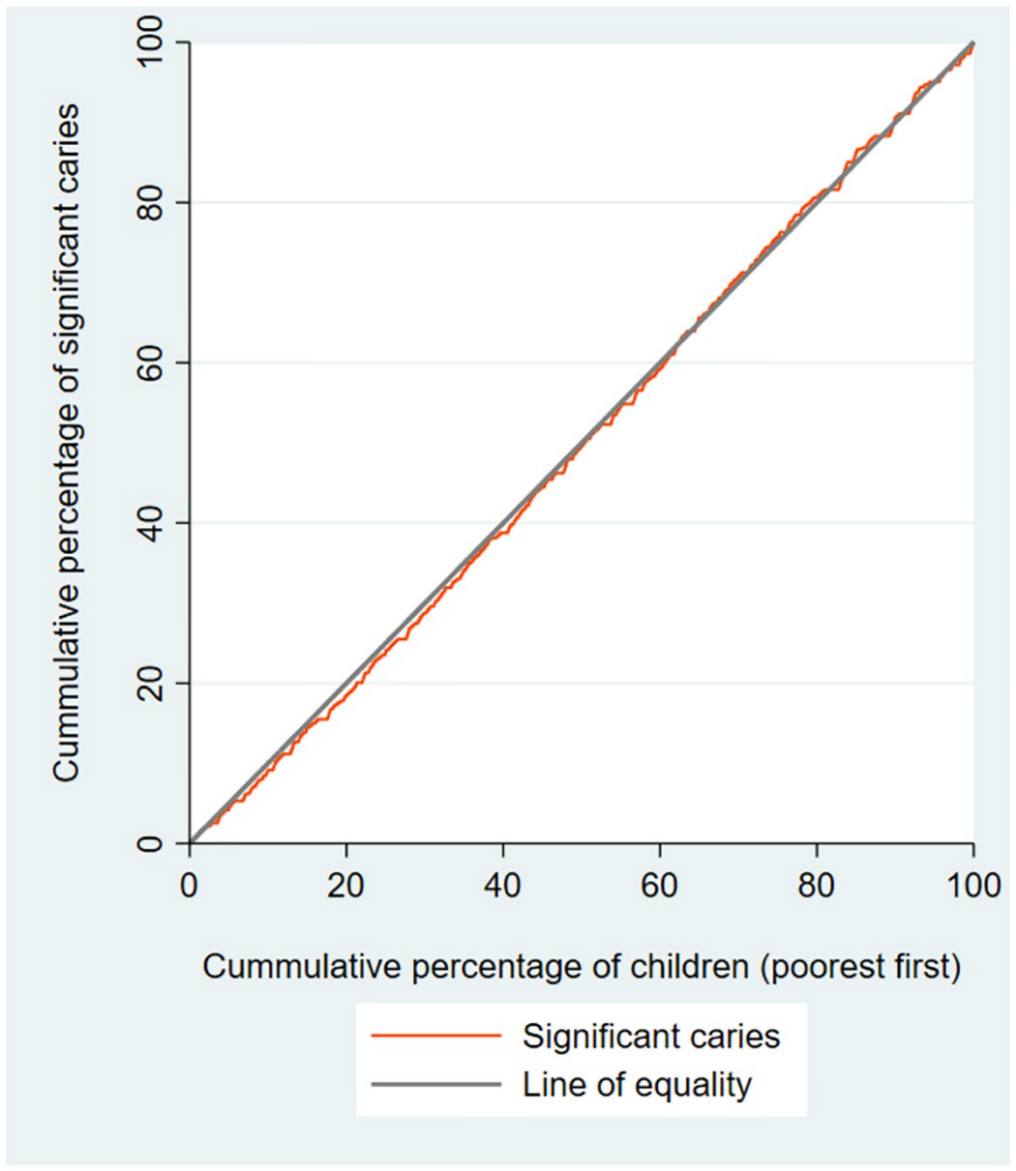
Concentration curves for significant caries among schoolchildren in Shahroud, Iran, 2015.

Table 2 presents summary statistics and concentration index of dental caries indices among schoolchildren. Overall, 71.4% of the schoolchildren had dft≥1, with the prevalence of DMFT≥1 and SC≥1 being 41.6% and 33.2%, respectively. The concentration indices for dft≥1, pdt≥1, and PMT≥1 were -0.136, -0.164, and -0.208, indicating a higher concentration of these outcomes among the low economic groups of children. However, the C for pft≥1 and PFT≥1 were 0.327 and 0.218, respectively, showing an economic inequality in favor of high-economic groups of children. (Figs 1–3).

**Table 2:** Summary statistics and economic inequality of dental health indices among schoolchildren in Shahroud, Iran, 2015.

Dental caries index	N (n)	Prevalence	95% CI	Concentration index	95% CI	P-value
dft≥1	4992 (3574)	71.36	(67.57, 75.15)	-0.136	(-0.167, -0.104)	<0.001
pdt≥1	4992 (3481)	69.26	(65.50, 73.03)	-0.164	(-0.194, -0.134)	<0.001
pft≥1	4992 (608)	13.70	(11.87, 15.54)	0.327	(0.292, 0.361)	<0.001
DMFT≥1	4992 (2088)	41.59	(39.08, 44.09)	0.003	(-0.018, 0.025)	0.875
PDT≥1	4992 (1863)	37.18	(34.80, 39.56)	-0.016	(-0.037, 0.006)	0.465
PMT≥1	4992 (154)	2.39	(1.85, 2.92)	-0.208	(-0.262, -0.153)	<0.001
PFT≥1	4992 (334)	7.04	(6.01, 8.08)	0.218	(0.179, 0.256)	<0.001
SC≥1	4992 (1664)	33.21	(30.87, 35.54)	0.007	(-0.016, 0.029)	0.761

dft: Primary decayed and filled teeth, pdt: Primary decayed teeth, pft: Primary filled teeth, DMFT: Permanent decayed, missing, and filled teeth, PDT: Permanent decayed teeth, PMT: Permanent missing teeth, PFT: Permanent filled teeth, SC: Significant caries, SD: Standard deviation, CI: Confidence interval

According to the results of decomposition analysis for primary teeth caries in Table 3, basic health insurance coverage and age were the two main contributors to the measured economic gradient in dft≥1 by 28.6% and 19.2%, respectively. Similarly, basic health insurance coverage and age had the largest contributions to the measured inequality in pdt≥1 at 25.7% and 16.6%, respectively. Economic status, residence in rural areas, mother’s education, and father’s education were the main factors that positively contributed to the measured inequality in pft≥1 by 80.5%, 26.5%, 21.9%, and 20.2%, respectively, while the opposite is true for age with a negative contribution of 18.9%.

**Table 3:** Decomposition of economic inequalities in schoolchildren’s primary teeth caries in Shahroud, Iran, 2015.

Characteristics	Marginal effect			Elasticity			C_k_	Absolute contribution to CI			Contribution (%)		
	dft≥1	pdt≥1	pft≥1	dft≥1	pdt≥1	pft≥1		dft≥1	pdt≥1	pft≥1	dft≥1	pdt≥1	pft≥1
Female gender	0.040	0.048	-0.007	0.030	0.037	-0.027	0.054	0.002	0.002	-0.001	-1.19	-1.23	-0.45
Age (RC: 6 years)													
7	0.002	-0.050	0.065	0.334*10^−3^	-0.011	0.071	-0.112	-0.037*10^−3^	0.001	-0.008	0.03	-0.73	-2.41
8	-0.030	-0.089	0.073	-0.008	-0.023	0.097	-0.055	0.428*10^−3^	0.001	-0.005	-0.32	-0.79	-1.64
9	-0.139	-0.178	0.026	-0.037	-0.049	0.037	-0.046	0.002	0.002	-0.002	-1.28	-1.40	-0.52
10	-0.296	-0.333	-0.056	-0.067	-0.077	-0.066	0.036	-0.002	-0.003	-0.002	1.77	1.69	-0.72
11	-0.438	-0.482	-0.119	-0.104	-0.118	-0.147	0.119	-0.012	-0.014	-0.018	9.14	8.56	-5.37
12	-0.587	-0.643	-0.209	-0.092	-0.104	-0.170	0.146	-0.013	-0.015	-0.025	9.90	9.22	-7.62
Sum								-0.026	-0.027	-0.060	19.24	16.56	-18.29
Rural residency	-0.022	-0.018	-0.205	-0.003	-0.003	-0.148	-0.586	0.002	0.001	0.087	-1.33	-0.90	26.53
Father education (RC = <5 years)													
5–12	-0.008	-0.009	0.056	-0.006	-0.007	0.227	-0.131	0.001	0.001	-0.030	-0.57	-0.57	-9.09
>12	-0.010	-0.031	0.105	-0.004	-0.012	0.205	0.466	-0.002	-0.006	0.096	1.32	3.38	29.32
Sum								-0.001	-0.005	0.066	0.75	2.80	20.22
Mother education (RC = <5 years)													
5–12	0.000	-0.003	0.049	0.000	-0.003	0.210	-0.046	-0.018*10^−3^	0.133*10^−3^	-0.010	0.01	-0.08	-2.95
>12	-0.017	-0.038	0.109	-0.005	-0.011	0.166	0.489	-0.002	-0.006	0.081	1.81	3.41	24.85
Sum								-0.002	-0.005	0.072	1.82	3.33	21.89
Father employment status (RC = Employed)													
Unemployed/retired	-0.024	-0.030	0.015	-0.003	-0.004	0.010	-0.073	0.220*10^−3^	0.286*10^−3^	-0.001	-0.16	-0.17	-0.22
Other	-0.050	-0.050	-0.043	-0.003	-0.003	-0.012	-0.264	0.001	0.001	0.003	-0.50	-0.44	0.94
Sum								0.001	0.001	0.002	-0.67	-0.61	0.72
Have a housekeeper mother	-0.001	-0.010	0.021	-0.001	-0.011	0.127	-0.251	0.267*10^−3^	0.003	-0.032	-0.20	-1.75	-9.74
Economic quintile (RC = 1^st^)													
2^nd^	0.017	0.019	0.029	0.004	0.005	0.040	-0.616	-0.003	-0.003	-0.025	2.02	2.01	-7.63
3^rd^	0.010	-0.005	0.100	0.003	-0.002	0.163	-0.108	-0.332*10^−3^	0.188*10^−3^	-0.018	0.24	-0.11	-5.39
4^th^	0.003	-0.013	0.119	0.001	-0.004	0.184	0.446	0.394*10^−3^	-0.002	0.082	-0.29	1.04	25.08
5^th^	-0.016	-0.031	0.140	-0.005	-0.010	0.223	1.000	-0.005	-0.010	0.223	3.65	6.05	68.39
Sum								-0.008	-0.015	0.263	5.62	8.98	80.45
Attend a private school	-0.004	-0.016	-0.009	-0.001	-0.002	-0.006	0.474	0.000	-0.001	-0.003	0.18	0.62	-0.91
Overweight/obesity	-0.089	-0.080	-0.034	-0.030	-0.028	-0.060	0.142	-0.004	-0.004	-0.009	3.19	2.42	-2.63
Have basic health insurance	-0.054	-0.057	-	-0.075	-0.082	-	0.516	-0.039	-0.042	-	28.57	25.73	-
Have supplementary health insurance	0.014	0.002	0.010	0.004	0.001	0.016	0.271	0.001	0.162*10^−3^	0.004	-0.80	-0.10	1.32
**Total observed**								**-0.078**	**-0.092**	**0.389**	**55.82**	**55.85**	**119.13**
**Residual**								**-0.057**	**-0.072**	**-0.062**	**44.18**	**44.15**	**-19.13**
**Total**								**-0.136**	**-0.164**	**0.327**	**100**	**100**	**100**

dft: Primary decayed and filled teeth, pdt: Primary decayed teeth, pft: Primary filled teeth, RC: Reference category

Table 4 represents the results of decomposition analysis for PMT≥1 and PFT≥1. Based on the findings, economic status, having a housekeeper mother, residing in rural areas, having basic health insurance coverage, mother’s education, and father’s education made the most positive contributions to the measured inequality in PMT≥1 by 45.4%, 42.4%, 37.8%, 35.1%, 21.3%, and 15.2%, respectively. Age had the most negative contribution of -19.3%. In the case of PFT≥1, economic status, age, and father’s education accounted for 76%, 25.4%, and 20.3% of the measured inequality, respectively.

**Table 4:** Decomposition of economic inequalities in schoolchildren’s permanent teeth caries in Shahroud, Iran, 2015.

Characteristics	Marginal effect		Elasticity		C_k_	Absolute contribution to CI		Contribution (%)	
	PMT≥1	PFT≥1	PMT≥1	PFT≥1		PMT≥1	PFT≥1	PMT≥1	PFT≥1
Female gender	0.001	-0.033	0.026	-0.249	0.054	0.001	-0.013	-0.68	-6.16
Age (RC: 6 years)									
7	-0.012	0.031	-0.074	0.065	-0.112	0.008	-0.007	-3.98	-3.34
8	0.002	0.084	0.015	0.218	-0.055	-0.001	-0.012	0.41	-5.52
9	0.004	0.106	0.034	0.288	-0.046	-0.002	-0.013	0.77	-6.14
10	0.019	0.128	0.129	0.294	0.036	0.005	0.011	-2.23	4.85
11	0.019	0.142	0.132	0.342	0.119	0.016	0.041	-7.58	18.74
12	0.020	0.158	0.094	0.250	0.146	0.014	0.037	-6.65	16.81
Sum						0.040	0.055	-19.25	25.40
Rural residency	0.032	-0.008	0.134	-0.011	-0.586	-0.078	0.007	37.77	3.04
Father education (RC = <5 years)									
5–12	-0.008	0.018	-0.183	0.142	-0.131	0.024	-0.019	-11.55	-8.53
>12	-0.011	0.035	-0.119	0.135	0.466	-0.056	0.063	26.77	28.82
Sum						-0.032	0.044	15.23	20.29
Mother education (RC = <5 years)									
5–12	0.003	-0.001	0.078	-0.005	-0.046	-0.004	0.000	1.73	0.11
>12	-0.010	0.010	-0.083	0.030	0.489	-0.041	0.014	19.61	6.66
Sum						-0.044	0.015	21.34	6.77
Father employment status (RC = Employed)									
Unemployed/retired	-0.006	0.006	-0.022	0.008	-0.073	0.002	-0.001	-0.76	-0.26
Other	0.003	-0.018	0.005	-0.010	-0.264	-0.001	0.003	0.59	1.18
Sum						0.000	0.002	-0.16	0.92
Have a housekeeper mother	0.010	-0.002	0.350	-0.027	-0.251	-0.088	0.007	42.42	3.15
Economic quintile (RC = 1^st^)									
2^nd^	-0.003	0.028	-0.025	0.077	-0.616	0.015	-0.047	-7.31	-21.79
3^rd^	0.001	0.044	0.012	0.141	-0.108	-0.001	-0.015	0.65	-6.99
4^th^	-0.004	0.053	-0.031	0.160	0.446	-0.014	0.071	6.70	32.67
5^th^	-0.010	0.051	-0.094	0.157	1.000	-0.094	0.157	45.39	72.13
Sum						-0.094	0.165	45.43	76.02
Attend a private school	0.005	0.000	0.018	0.000	0.474	0.008	0.000	-4.00	0.09
Overweight/obesity	-0.007	-0.006	-0.075	-0.020	0.142	-0.011	-0.003	5.12	-1.27
Have basic health insurance	-0.003	-	-0.141	-	0.516	-0.073	-	35.12	-
Have supplementary health insurance	0.003	0.003	0.023	0.010	0.271	0.006	0.003	-2.98	1.22
**Total observed**						**-0.364**	**0.282**	**175.35**	**129.46**
**Residual**						**0.156**	**0.045**	**-75.35**	**-29.46**
**Total**						**-0.208**	**0.327**	**100**	**100**

PMT: Permanent missing teeth, PFT: Permanent filled teeth, RC: Reference category

## 4. Discussion

The present study has investigated economic inequalities in health indices of children’s primary and permanent teeth. The findings of this study are interpreted in two sections, as follows:

### 4.1. Economic inequalities in primary teeth caries

The study findings showed that dft≥1 was more concentrated among children from low-economic backgrounds, and while untreated caries in the primary dentition (pdt≥1) was more concentrated among low-economic children, high-economic children benefited from carious teeth restoration in their primary dentition (pft≥1). The contribution of various factors to the observed inequalities in primary teeth health indices was discussed as below:

#### 4.1.1. Health insurance coverage

According to the results of decomposition analyses, basic health insurance coverage is the main factor that had the largest contribution to the measured pro-rich economic gradients in the distribution of dft≥1 and pdt≥1. Basic health insurance programs cover various dental services, including dental examinations, radiography, tooth extraction, surgical removal of impacted and semi-impacted teeth, supragingival scaling and oral hygiene instruction, subgingival scaling (only for people with the age of >12 years), tooth polishing, and restoration of first molars for 6- to12-year-old children [30]. These programs generally cover a small part of the costs of dental health services in the country. As it is reported by the Iran Dental Association, only 11% of dental costs were covered by the health insurance programs, and out of pocket payments accounted for 89% of these costs [31]. Our research indicates that children with basic health insurance had lower rates of dft≥1 and pdt≥1, although this difference was not statistically significant. Despite dental service costs being generally high for insured households in Iran, basic health insurance programs do provide some coverage for children, potentially reducing the prevalence of tooth decay. Furthermore, our findings revealed that basic health insurance coverage was more prevalent among high-economic households compared to low-economic ones (as indicated by a concentration index of 0.516 in Table 3). This significant inequality in distribution of insurance coverage could be the main reason for the contribution of this factor to the measured economic inequality in children’s tooth decay.

On the other hand, it was expected that the higher concentration of supplementary health insurance coverage among children from higher economic backgrounds (the concentration index of supplementary health insurance was 0.271) would exacerbate the measured pro-rich inequality in children’s dental restoration. However, as our study findings revealed, the impact of supplementary health insurance on dental restoration inequality was insignificant, and supplementary health insurance coverage made a minimal contribution to the observed inequality in pft≥1. One possible explanation for these findings is that in Iran, most supplementary health insurance programs did not provide sufficient coverage for household dental services. Dental services are a crucial factor influencing Iranian households’ exposure to catastrophic health expenses, and low-income households are less likely to face these costs because they do not utilize these services [32]. These findings suggest that expanding basic and supplementary health insurance coverage for the poor, along with adequate coverage for dental health expenses, could reduce inequalities in children’s primary tooth decay.

#### 4.1.2. Socioeconomic factors

The findings of our research showed that the economic status of households did not significantly contribute to the inequality in dft≥1 and pdt≥1. However, 80.5% of the observed inequality in pft≥1 was attributed to the economic status of households. Offering oral health education and check-ups at health houses (primary healthcare facilities providing essential medical services to rural communities), as well as dental treatment services at rural and urban health centers [33] under basic health insurance programs, could help eliminate financial barriers for poor children in accessing these services. However, the coverage of dental restoration services by health insurance schemes was inadequate, forcing families to choose between these services and essential household needs, potentially leading to delayed treatment of decayed teeth or opting for lower-cost alternatives like tooth extraction. Poor households have to spend a large proportion of their income on dental health services as the out of pocket expenses.

Residing in rural areas and having lower-educated parents are the next socioeconomic factors that account for the observed inequality in pft≥1. Other studies have shown more primary teeth caries among rural children [14, 34–36] and those with low-educated parents [37, 38], and found a negative association of filling the carious teeth with mother’ low education [39]. In our study, there is no difference between urban and rural children’s primary teeth caries, however, it seems that poor rural children and those with low-educated parents had poor dental hygiene behaviors, such as brushing and regular dental check-ups [40–42], which could lead to more severe tooth decay. These children also have less financial and physical access to expensive dental restoration services, which were not provided in rural areas [33].

#### 4.1.3. Age

In this study, we found a negative association between children’s age and their primary tooth decay. This finding is expected due to the transition from primary teeth to permanent ones as children grow older, aligning with findings from other studies [36, 43]. On the other hand, the Cs of age groups in Table 3 show that older children had better economic status. Specifically, older children from higher economic backgrounds exhibited lower levels of primary tooth decay. That is why higher ages positively contributed to the measured inequalities in dft≥1 and pdt≥1, and negatively contributed to the pft≥1 inequality. This means that the concentration of untreated primary teeth caries among low-economic children was higher than among high-economic older children. These findings underscore the importance of providing adequate dental care for primary teeth in young children from disadvantaged backgrounds. Therefore, prioritizing the oral health of underprivileged children and addressing tooth decay at an early age can significantly reduce inequalities in primary tooth decay.

### 4.2. Economic inequalities in permanent teeth caries

For permanent teeth, the measured concentration indices suggested that the DMFT≥1, SC≥1, and PDT≥1 did not differ significantly between the low- and high-economic children. Some studies have not found a significant relationship between households’ economic status and permanent teeth caries [44, 45], while in some others, economic status has a positive or negative association with children’s dental caries [13, 41, 46]. One explanation for our findings could be that high-economic households, because of their greater financial affordability, can provide more access to oral hygiene tools, including toothpaste, toothbrush and floss [47], as well as, dental check-up services [14, 41], which can be effective in preventing children’s tooth decay [48]. However, more financial ability can lead their children to consume high-calorie and sugary food and drinks, which could have a contributory effect on children’s tooth decay [49, 50].

Another explanation for the equal distribution of DMFT≥1 among the low- and high-economic children is that although DMFT≥1 and PDT≥1 did not differ between the low- and high-economic children, economic status have an association with the treatment methods of dental caries. Extracting the carious permanent teeth (PMT≥1) was more concentrated among low-economic children, and PFT≥1 was more concentrated among the high-economic children, indicating they benefited more from restoration services.

#### 4.2.1. Health insurance coverage

The study findings showed that the prevalence of PMT≥1 is significantly lower among children who had basic health insurance coverage, which resulted in more concentration of permanent tooth extraction among low-economic children with no basic health insurance coverage. This highlights the importance of basic health insurance in addressing the need of low-economic children for prevention and check-up services and the need to expand insurance coverage for these children.

#### 4.2.2. Socioeconomic factors

According to decomposition analyses, a significant portion of the measured inequality in PMT≥1 was explained by socioeconomic factors such as household’s poor economic status, having a housekeeper mother, residing in rural areas, and parents’ low education, all more common among low-economic children. For PFT≥1, 76% of the observed inequality was attributable to the households’ economic status, and father education was the second main contributing socioeconomic factor. Other studies have shown similar results; For example, in the study conducted by Bobu et al, the prevalence of missing teeth in the children’s mixed dentition had a negative association with the households’ income, while the opposite finding was observed for the filling teeth [51]. Findings of studies indicated that children with a housekeeper mother were more likely to have dental caries and untreated tooth decay than those with an employed mother [49, 52, 53]. Additionally, extracting carious permanent teeth was more prevalent among rural children than urban ones [16, 54], and children with low-educated parents had more extracted teeth [42, 55] and fewer restored teeth [42].

Low-economic and rural households have limited financial and physical access to dental restoration services, leading to delays in timely filling of carious permanent teeth. Unmet needs for restoration services could worsen tooth decay severity and increase the likelihood of extraction among these children. Furthermore, as indicated in other studies, lower education and awareness, along with limited financial resources to provide a healthy diet and dental health services by low-SES parents, could contribute to their children’s higher rates of tooth decay [14, 41, 47, 48, 56] and consequently, more reliance on tooth extraction over restoration services [12].

#### 4.2.3. Age

Age was another factor that had a substantial contribution to the measured inequalities in permanent missing and filled teeth. The prevalence of permanent missing and filled teeth had positive associations with the age of children. Similar results have been found in other studies [16, 36, 43]. The higher prevalence of extracted or filled teeth in older age groups is attributed to the increase in the number of permanent teeth and the progression of tooth decay over time. Specifically, young children from low-economic backgrounds have fewer extracted and restored permanent teeth, while older children from high-economic backgrounds experience more extractions and restorations. These findings suggest that providing dental restoration services at a younger age could help reduce the economic inequalities in children’s current dental health status.

### 4.3. Strength and limitations

To our knowledge, this study was the first to investigate economic inequalities in primary and permanent teeth caries among schoolchildren in Iran. Further analysis revealed no significant differences in dental indices between the included (4992) and excluded (585) students, allowing for generalization of the study results to the target population. One limitation to note is the lack of data on parental and child awareness of dental hygiene, children’s dietary habits, dental self-care behaviors, and utilization of dental health services, which could help explain the observed economic gradients in children’s dental caries. Future studies are recommended to explore the impact of these factors on the identified inequalities in children’s dental caries. This study did not assess the agreement between two dentists in dental examinations. However, the dentists underwent training with a standardized procedure for dental assessments following WHO guidelines. Additionally, given the cross-sectional nature of the data utilized in the study, caution is advised in making causal interpretations.

## 5. Conclusions

Pro-rich economic inequalities were observed in children’s primary and permanent teeth caries. Thus, government interventions to reduce these inequalities should aim to expand the coverage of basic and supplementary health insurance in line with increasing the coverage of dental health costs in these plans, training and providing access to required dental health services for low-socioeconomic children, including the poor, rural, and those who have low-educated parents and a housekeeper mother, especially at younger ages.

## Supporting information

S1 Checklist STROBE statement—Checklist of items that should be included in reports of observational studies. (DOCX)

## Abbreviations

C: Concentration index
CI: Confidence Interval
Cov: Covariance
dft: Primary decayed and filled teeth
DMFT: Decayed, Missing and Filled Teeth
PCA: Principal Component Analysis
PDT: Permanent Decayed Teeth
pdt: Primary decayed teeth
PFT: Permanent filled teeth
pft: Primary filled teeth
PMT: Permanent missing teeth
RC: Reference Category
SC: Significant caries
SiC: Significant Caries Index
SSCECS: Shahroud School Children Eye Cohort Study
VIF: Variance Inflation Factor
WHO: World Health Organization
